# Correction: Schavkan, A., *et al.* Number Concentration of Gold Nanoparticles in Suspension: SAXS and spICPMS as Traceable Methods Compared to Laboratory Methods. *Nanomaterials* 2019, *9*, 502

**DOI:** 10.3390/nano9081060

**Published:** 2019-07-24

**Authors:** Alexander Schavkan, Christian Gollwitzer, Raul Garcia-Diez, Michael Krumrey, Caterina Minelli, Dorota Bartczak, Susana Cuello-Nuñez, Heidi Goenaga-Infante, Jenny Rissler, Eva Sjöström, Guillaume B. Baur, Konstantina Vasilatou, Alexander G. Shard

**Affiliations:** 1 Physikalisch–Technische Bundesanstalt (PTB), 10587 Berlin, Germany; 2 National Physical Laboratory (NPL), Middlesex TW11 0LW, UK; 3 LGC Limited, Teddington TW11 0LY, UK; 4 RISE Research Institutes of Sweden AB (SP), 11428 Stockholm, Sweden; 5 Federal Institute of Metrology (METAS), 3003 Bern-Wabern, Switzerland

The authors wish to make the following corrections to this paper [1]:

Reference 17 should be: Cuello-Nunez, S.; Bartczak, D.; Goenaga-Infante, H. Achieving SI traceability for number concentration of inorganic nanoparticles using spICP-MS without reference nanomaterials. Unpublished work, 2019.

The authors would like to apologize for any inconvenience caused to the readers by these changes.
